# Effects of the glucagon-like peptide-1 receptor agonist liraglutide in juvenile transgenic pigs modeling a pre-diabetic condition

**DOI:** 10.1186/s12967-015-0431-2

**Published:** 2015-02-25

**Authors:** Elisabeth Streckel, Christina Braun-Reichhart, Nadja Herbach, Maik Dahlhoff, Barbara Kessler, Andreas Blutke, Andrea Bähr, Nicole Übel, Matthias Eddicks, Mathias Ritzmann, Stefan Krebs, Burkhard Göke, Helmut Blum, Rüdiger Wanke, Eckhard Wolf, Simone Renner

**Affiliations:** Chair for Molecular Animal Breeding and Biotechnology, Gene Center, LMU Munich, Munich, Germany; Laboratory for Functional Genome Analysis (LAFUGA), Gene Center, LMU Munich, Munich, Germany; Institute of Veterinary Pathology, Center for Clinical Veterinary Medicine, LMU Munich, Munich, Germany; Clinic for Swine, Center for Clinical Veterinary Medicine, LMU Munich, Munich, Germany; Department of Internal Medicine II, Clinical Center of the LMU Munich, Campus Großhadern, Munich, Germany; Gene Center, LMU Munich, Feodor-Lynen-Str. 25, D-81377 Munich, Germany

**Keywords:** Transgenic pig model, Type 2 diabetes, GIP receptor, GLP1 receptor agonist, Incretin-based therapeutics, Liraglutide, Adolescents, Beta-cell mass

## Abstract

**Background:**

The glucagon-like peptide-1 receptor (GLP1R) agonist liraglutide improves glycemic control and reduces body weight of adult type 2 diabetic patients. However, efficacy and safety of liraglutide in adolescents has not been systematically investigated. Furthermore, possible pro-proliferative effects of GLP1R agonists on the endocrine and exocrine pancreas need to be further evaluated. We studied effects of liraglutide in adolescent pigs expressing a dominant-negative glucose-dependent insulinotropic polypeptide receptor (GIPR^dn^) in the beta-cells, leading to a pre-diabetic condition including disturbed glucose tolerance, reduced insulin secretion and progressive reduction of functional beta-cell mass.

**Methods:**

Two-month-old GIPR^dn^ transgenic pigs were treated daily with liraglutide (0.6-1.2 mg per day) or placebo for 90 days. Glucose homeostasis was evaluated prior to and at the end of the treatment period by performing mixed meal and intravenous glucose tolerance tests (MMGTT and IVGTT). Finally animals were subjected to necropsy and quantitative-stereological analyses were performed for evaluation of alpha- and beta-cell mass, beta-cell proliferation as well as acinus-cell proliferation.

**Results:**

MMGTT at the end of the study revealed 23% smaller area under the curve (AUC) for glucose, a 36% smaller AUC insulin, and improved insulin sensitivity, while IVGTT showed a 15% smaller AUC glucose but unchanged AUC insulin in liraglutide- vs. placebo-treated animals. Liraglutide led to marked reductions in body weight gain (-31%) and food intake (-30%) compared to placebo treatment, associated with reduced phosphorylation of insulin receptor beta (INSRB)/insulin-like growth factor-1 receptor beta (IGF1RB) and protein kinase B (AKT) in skeletal muscle. Absolute alpha- and beta-cell mass was reduced in liraglutide-treated animals, but alpha- and beta-cell mass-to-body weight ratios were unchanged. Liraglutide neither stimulated beta-cell proliferation in the endocrine pancreas nor acinus-cell proliferation in the exocrine pancreas, excluding both beneficial and detrimental effects on the pig pancreas.

**Conclusions:**

Although plasma liraglutide levels of adolescent transgenic pigs treated in our study were higher compared to human trials, pro-proliferative effects on the endocrine or exocrine pancreas or other liraglutide-related side-effects were not observed.

**Electronic supplementary material:**

The online version of this article (doi:10.1186/s12967-015-0431-2) contains supplementary material, which is available to authorized users.

## Background

GLP1R agonists represent a promising group of incretin-based therapeutics for type 2 diabetes [1]. Liraglutide is approved for the use in adult type 2 diabetic patients [2] and was shown to reduce body weight and improve glycemic control to a greater extent than exenatide [3-5]. Since the prevalence of type 2 diabetes is steadily increasing among adolescents while only few approved therapeutics exist, liraglutide can possibly address the unmet need for treatment of this group of patients [6]. However, effects of liraglutide on adolescents have not been systematically studied. The use of an adolescent animal model is important to gain insight into efficacy and safety of liraglutide in adolescent patients but also to evaluate possible pro-proliferative effects of incretin-based therapies in the endocrine and exocrine pancreas at a young age with presumably high cell proliferation capacity [7,8]. So far, studies in non-rodent species are rare but consistently showed no positive effect of liraglutide treatment on endocrine cell mass and proliferation rate [9-11]. Studies in numerous different rodent models that evaluated the effect of the GLP1R agonist liraglutide on beta-cell mass, proliferation and apoptosis showed variable results. While some of them revealed a substantial increase in beta-cell mass up to almost 40% and positive effects on beta-cell proliferation rate, others detected unaltered or even reduced beta-cell mass and/or proliferation rate [12-19]. But it has to be taken into account that the rodent pancreas has higher capacity for beta-cell proliferation compared to the human pancreas [20,21]. Therefore, findings in rodents do not necessarily reflect effects of liraglutide on human beta-cells. Also, rodent studies suggested a pro-proliferative influence of GLP1R agonists on the exocrine pancreas [22-24], and a recent study even reported marked expansion of exocrine tissue and a potential risk for neuroendocrine tumors in human pancreata after incretin therapy [25]. As results from rodent studies are inconsistent, studies in non-rodent species as well as cadaveric organs of patients with incretin therapy are rare, and the *in vivo* monitoring of cell proliferation in human pancreata is still challenging, studies with large animal models may improve the understanding of GLP1 analogue action on cell proliferation in the pancreas and associated side-effects. The pig is physiologically similar to human and thus has the potential to bridge the gap between rodent models and human patients [26,27]. Transgenic pigs expressing a GIPR^dn^ in their beta-cells mimic important aspects of human pre-diabetes: impaired function of the incretin hormone GIP, disturbed glucose tolerance, reduced insulin secretion, and progressive reduction of beta-cell mass [7,28]. Since the function of the GLP1R is not disturbed in this pig model [7], we performed a treatment trial with the GLP1R agonist liraglutide in order to address the following questions: 1.) How does liraglutide physiologically affect adolescent organisms? 2.) Can liraglutide maintain or expand physiological beta-cell mass? 3.) Does liraglutide induce cell proliferation in the endocrine or exocrine pancreas?

## Methods

### Animals and study design

**Figure 1:**
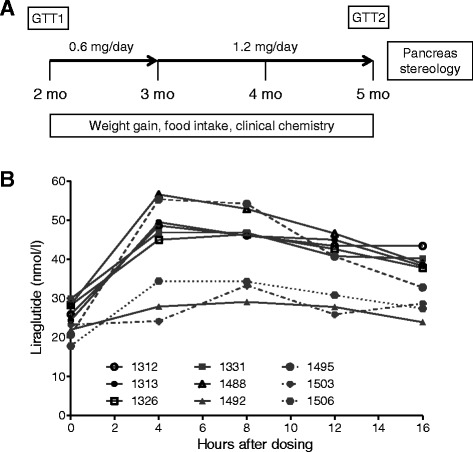
**Study design and plasma liraglutide levels. (A)** Study outline/liraglutide dosage regimen, mo = months of age, GTT = glucose tolerance testing including mixed meal glucose tolerance test (MMGTT) and intravenous glucose tolerance test (IVGTT). **(B)** Plasma liraglutide levels at the end of the treatment period in non-fasted GIPR^dn^ transgenic pigs after 1.2 mg liraglutide injection; 0 hours = point of liraglutide administration.

Eighteen hemizygous GIPR^dn^ transgenic pigs [7] were randomly assigned to liraglutide treatment using prefilled pens (Victoza®, 6 mg/ml, Novo Nordisk A/S) or placebo treatment (0.9% NaCl, B. Braun), injected subcutaneously once daily for 90 days. Liraglutide doses (0.6-1.2 mg per day, Figure 1A) were based on human dosages adjusted for pig body weight. Pigs were housed in planar single pens with straw litter and had *ad libitum* access to water and a standard pig diet (see Additional file 1: Table S1 for diet composition). All animal experiments were performed in accordance with the German Animal Welfare Act and approved by the responsible animal welfare authority. Pigs were treated with liraglutide (n = 9; 5 females, 4 males) or placebo (n = 9; 5 females, 4 males) from 2 to 5 months of age. At the age of 2 months untreated GIPR^dn^ transgenic pigs show disturbed oral glucose tolerance and delayed insulin secretion, but unaltered total beta-cell volume [7]. The physiological characteristics of the pigs prior to liraglutide/placebo treatment are summarized in Table 1.

**Table 1:** **Physiological characteristics of GIPR** ^**dn**^ **transgenic pigs one week prior to the start of liraglutide/placebo treatment**

**Parameter**	**Control group**	**Treatment group**	**P**
	**(Placebo)**	**(Liraglutide)**	
	**n = 9**	**n = 9**	
	**4 m, 5 f**	**4 m, 5 f**	
Age [days]	60 ± 0.14	60 ± 0.14	1.00
Body weight [kg]	15 ± 1.00	16 ± 0.73	0.41
MMGTT			
AUC glucose	17839 ± 682	18375 ± 881	0.80
AUC insulin	2956 ± 187	3296 ± 261	0.30
IVGTT			
AUC glucose	13015 ± 592	12821 ± 458	0.86
AUC insulin	719 ± 69	862 ± 75	0.08

m = male, f = female, MMGTT = mixed meal glucose tolerance test, IVGTT = intravenous glucose tolerance test, AUC = area under the curve. Data are means ± SEM.

### Measurements of body weight and food intake

Body weight was determined once weekly to the nearest 0.5 kg and food intake was recorded per 3 days. Feeding efficiency corresponds to total body weight gain divided by entire food intake during the treatment period. Animals were monitored daily for side effects.

### Clinical chemistry

Blood samples were taken every month after an 18-hour fasting period. Serum was separated by centrifugation and analyzed for standard clinical-chemical parameters using an Autoanalyzer Hitachi 911® and adapted reagents from Roche Diagnostics GmbH.

### Glucose tolerance test

A mixed meal glucose tolerance test (MMGTT) as well as an intravenous glucose tolerance test (IVGTT) was performed prior to and at the end of the 90-day treatment period after an 18-hour fasting period. Central venous catheters (Cavafix® Certo®, B. Braun) were surgically inserted into the external jugular vein and the tests were performed as described previously [7]. For the MMGTT 2 g glucose/kg body weight mixed with 50-200 g pig fodder (Deuka porfina U, Deuka) depending on the body weight of the pigs were administered. For the IVGTT a bolus injection of concentrated 50% glucose solution (0.5 g per kg body weight) was administered through the central venous catheter. Liraglutide/placebo was administered 3 hours before the test. Plasma glucose/insulin levels were measured as described previously [7] and plasma glucagon levels were determined by radioimmunoassay (Millipore). The net glucose elimination rate after glucose injection was calculated as the slope for the interval 1-30 minutes after glucose injection of the logarithmic transformation of the individual plasma glucose values [29].

### Plasma liraglutide levels

At the end of the treatment period, plasma liraglutide levels were determined in non-fasted animals after injection of 1.2 mg liraglutide. Blood samples were collected at 0, 4, 8, 12 and 16 hours relative to liraglutide administration in EDTA monovettes pre-treated with diprotin A (Sigma-Aldrich) and aprotinin (Roth). Liraglutide levels were measured by Novo Nordisk A/S, using an in-house luminescence oxygen channeling immunoassay validated for pig plasma.

### Insulin sensitivity

To evaluate insulin sensitivity the HOMA-IR and the insulin sensitivity index according to Matsuda [ISI_(Matsuda)_] were calculated [30].

### Necropsy and pancreas sampling

Following the treatment period pigs were euthanized and selected organs were weighed. A subgroup of liraglutide- (n = 5) and placebo-treated animals (n = 4) was fasted for 12 hours before necropsy to assess effects on gastric emptying. The pancreas was explanted *in toto*, connective tissue was separated and the organ was weighed. Twenty pancreas samples per animal were chosen by systematic random sampling and routinely processed for paraffin histology [7]. Skeletal muscle samples were taken from the *biceps brachii* muscle, shock frozen, and stored at -80°C.

### Immunohistochemistry and quantitative-stereological analyses

For histological evaluation and quantitative-stereological analyses sections of 20 different pancreas positions were investigated. Sections were stained with hematoxylin and eosin (H&E) for detailed histological assessment by two independent pathologists. Insulin-containing cells were stained using guinea pig anti-porcine insulin antibodies (1:1000, Dako Cytomation) and goat anti-guinea pig IgG (1:100, Southern Biotech) with Vector® Red (Vector Laboratories Inc.) as chromogen. Glucagon-containing cells were stained with rabbit anti-glucagon antibodies (1:300, Dako) and goat anti-rabbit IgG (1:100, Dako) visualized with DAB (Biotrend). Mayer’s hemalum (Applichem GmbH) served as counterstain. Quantification of alpha-/beta-cell volume was carried out using the Visiomorph™ image analysis system (Visiopharm A/S). Volume densities [Vv_(alpha-cell/Pan)_, Vv_(beta-cell/Pan)_] as well as total alpha-/beta-cell volume [V_(alpha-cell, Pan)_, V_(beta-cell, Pan)_] were determined as described previously [7] and the corresponding alpha-/beta-cell volume-to-body weight ratios were calculated. Proliferation of beta-cells/acinus-cells was determined in sections co-stained with mouse anti-human Ki67 antibodies (1:8, Dako) and goat anti-mouse IgG (1:20, Dako) with BCIP/NBT Substrate Kit (Vector Laboratories Inc.) as chromogen as well as guinea pig anti-porcine insulin antibodies (1:1000, Dako) and rabbit anti-guinea pig IgG (1:100, Southern Biotech) with DAB as chromogen and Mayer’s hemalum as counterstain. Sections were systematically sampled using the stereology software newCast (Visiopharm) and cell proliferation was quantified as the number of Ki67+ labeled beta-cell/acinus-cell nuclei profiles divided by the total number of beta-cell/acinus-cell nuclei profiles counted and expressed as the number of Ki67+ labeled cell nuclei profiles per 10^5^ cell nuclei profiles.

### Western blot analyses

For protein extraction, muscle tissue was homogenized in Laemmli-extraction buffer, and the protein content was determined by the bicinchoninic acid protein assay. 40 μg of total protein was separated by SDS-PAGE and transferred to PDVF membranes (Millipore) by electroblotting. Membranes were washed in Tris-buffered saline solution with 0.1% Tween-20 and blocked in 5% w/v fat-free milk powder (Roth) for 1 h. Then membranes were washed again and incubated in 5% w/v BSA (Roth) solution with the appropriate primary antibody (Additional file 2: Table S2) overnight at 4°C. After washing, membranes were incubated in 5% w/v fat-free milk powder with a secondary antibody (donkey anti-rabbit; 1:2000; GE-Healthcare) for 1 h. Bound antibodies were detected using an enhanced chemiluminescence detection reagent (ECL Advance Western Blotting Detection Kit, GE Healthcare) and appropriate films (GE Healthcare). After detection, membranes were stripped and incubated with a second antibody. Band intensities were quantified using the ImageQuant software package (GE Healthcare).

### Statistics

All data are presented as means ± SEM. For statistical analysis, longitudinal data were square-root transformed to approximate normal distribution. Since significant deviations from normal distribution were only rarely detected (Shapiro-Wilk test in the Univariate Procedure; SAS 8.2), transformed data for body weight, food intake, glucose, insulin and glucagon levels were evaluated by ANOVA (Linear Mixed Models; SAS 8.2) taking the fixed effects of Group (liraglutide-/placebo-treated), Time (relative to glucose administration/treatment duration), and the interaction Group*Time into account. Transformed data of clinical-chemical analyses were evaluated by ANOVA (General Linear Models; SAS 8.2) taking the fixed effects of Group, Age, and the interaction Group*Age into account. AUC insulin/glucose was calculated using GraphPad Prism® software (version 5.02). AUCs and all remaining parameters were tested for significance by Mann-Whitney-*U*-test using GraphPad Prism® software. Results of Western blot analyses were related to the mean value of the placebo group (set to 1) and presented as box-plots with median. P values less than 0.05 were considered significant. Net glucose elimination rates [29] were calculated and slopes were compared using GraphPad Prism® software (version 5.02).

## Results

### Plasma liraglutide levels

Individual plasma liraglutide levels ranged between 17.8 and 56.6 nmol/l (Figure 1B). The highest mean plasma liraglutide levels were determined in the plasma sample 8 hours after application.

### Reduced body weight gain

**Figure 2:**
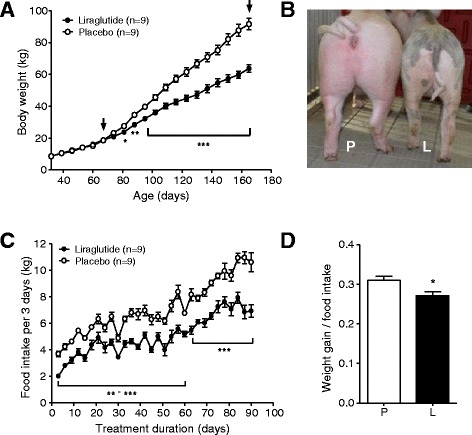
**Body weight gain and food intake in liraglutide- and placebo-treated GIPR** ^**dn**^ **transgenic pigs. (A)** Body weight gain of GIPR^dn^ transgenic pigs during pre-treatment and treatment period, black arrows indicate beginning/end of liraglutide/placebo administration. **(B)** Representative physical appearance of two female littermates (L = liraglutide-treated pig, P = placebo-treated pig) during the last third of the treatment period. **(C)** Food intake and **(D)** feeding efficiency of liraglutide- vs. placebo-treated GIPR^dn^ transgenic pigs during the treatment period, n = number of animals investigated. Data are means ± SEM. For statistical analysis (ANOVA; Linear Mixed Models; SAS 8.2), data were square-root transformed. *: p < 0.05, **: p < 0.01, ***: p < 0.001.

Liraglutide-treated GIPR^dn^ transgenic pigs gained distinctly less body weight compared to their placebo-treated counterparts, resulting in a 31% reduced body weight (63.7 ± 2.4 kg vs. 91.6 ± 3.7 kg; p < 0.001) at the end of the 90-day treatment period (Figure 2A). In addition to the reduced body weight, liraglutide-treated pigs appeared slightly smaller in size and shape than the corresponding placebo-treated pigs (for representative examples, see Figure 2B).

### Reduced food intake and feeding efficiency

Generally, pigs showed a steadily increasing food intake (Figure 2C). Nevertheless, liraglutide caused an immediate and sustained significant decrease in food intake compared to placebo, with mean differences of 30% (Figure 2C). Additionally, feeding efficiency was significantly reduced in liraglutide- vs. placebo-treated GIPR^dn^ transgenic pigs (Figure 2D).

### Health status and clinical-chemical parameters

During the treatment period, the general condition of all animals was undisturbed and adverse gastrointestinal effects like vomiting, obstipation or diarrhea were not observed. Clinical-chemical analyses revealed no differences between liraglutide- and placebo-treated GIPR^dn^ transgenic pigs in most parameters measured. However, liraglutide-treated pigs showed significantly lower serum levels of total protein (-8.6 ± 0.2%, p < 0.001), albumin (-12.7 ± 1.7%, p < 0.05) and phosphate (-11.8 ± 4.7%, p < 0.01) compared to their placebo-treated counterparts. Additionally, alkaline phosphatase (AP) activity was significantly increased in liraglutide-treated pigs (+10.4 ± 0.5%, p < 0.01) (see Additional file 3: Table S3).

### Improved oral glucose tolerance, decreased insulin secretion, and improved insulin sensitivity

**Figure 3:**
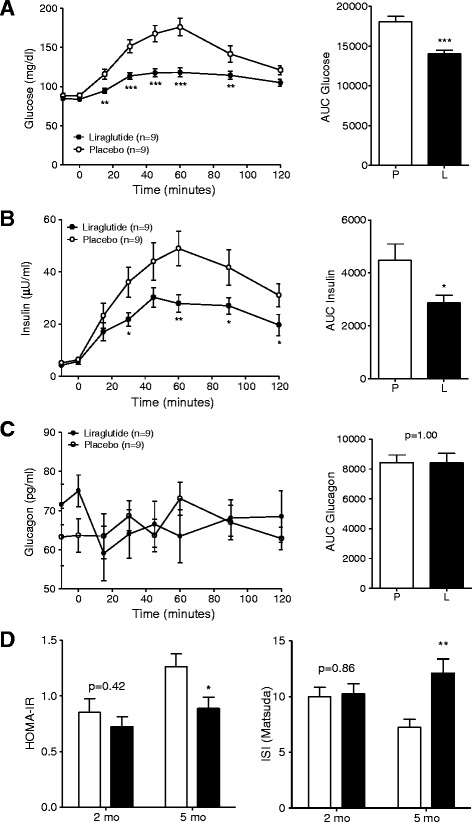
**Glucose control and insulin sensitivity during MMGTT in liraglutide- and placebo-treated GIPR** ^**dn**^ **transgenic pigs. (A)** Plasma glucose levels and AUC glucose as well as **(B)** plasma insulin levels and AUC insulin and **(C)** plasma glucagon levels and AUC glucagon during MMGTT in 18-hour fasted GIPR^dn^ transgenic pigs after the 90-day treatment period, 0 min. = point of glucose administration. **(D)** Insulin sensitivity indices prior to (2-month-old pigs) and after the treatment period (5-month-old pigs), mo = months of age, n = number of animals investigated. Data are means ± SEM. For statistical analysis (ANOVA; Linear Mixed Models; SAS 8.2), data were square-root transformed. *: p < 0.05, **: p < 0.01, ***: p < 0.001.

**Figure 4:**
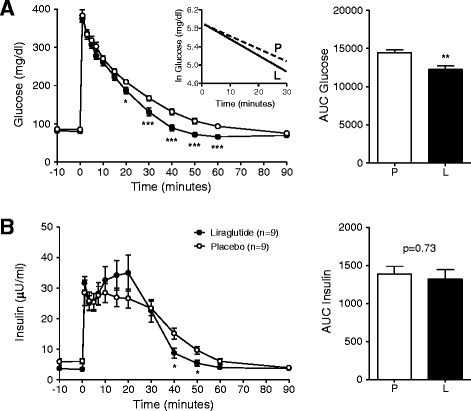
**Glucose control during IVGTT in liraglutide- and placebo-treated GIPR** ^**dn**^ **transgenic pigs. (A)** Plasma glucose levels and AUC glucose as well as **(B)** plasma insulin levels and AUC insulin during IVGTT in 18-hour fasted GIPR^dn^ transgenic pigs after the 90-day treatment period, 0 min. = point of glucose administration, n = number of animals investigated. Data are means ± SEM. For statistical analysis (ANOVA; Linear Mixed Models; SAS 8.2), data were square-root transformed. *: p < 0.05, **: p < 0.01, ***: p < 0.001. The insert in panel **(A)** shows the net glucose elimination rate after glucose injection as calculated as the slope for the interval 1–30 min after glucose injection of the logarithmic transformation of the individual plasma glucose values. The slopes of the net glucose elimination rate for the placebo (P: -0.0274 ± 0.0014) and liraglutide (L: -0.0355 ± 0.0011) groups were significantly different (p <0.001).

GIPR^dn^ transgenic pigs allocated to the liraglutide and placebo treatment groups did not show significant differences in glucose tolerance and insulin secretion during MMGTT and IVGTT performed prior to treatment (Table 1). However, during the second MMGTT at the end of the 90-day treatment period, plasma glucose increased much less in liraglutide- than in placebo-treated pigs, resulting in a 23% smaller AUC glucose (p < 0.001, Figure 3A). Accordingly, AUC insulin of liraglutide-treated pigs was 36% reduced (p < 0.05; Figure 3B) as compared to their placebo-treated counterparts. Glucagon levels during the MMGTT were not significantly different between liraglutide- and placebo-treated animals (Figure 3C). Moreover, liraglutide markedly improved insulin sensitivity, as shown by significantly reduced HOMA-IR and increased ISI_(Matsuda)_ after the treatment period (Figure 3D). The IVGTT at the end of the treatment period revealed a 15% decreased AUC glucose in liraglutide- vs. placebo-treated animals (p < 0.01; Figure 4A). The slopes of the net glucose elimination rate for the placebo (P: -0.0274 ± 0.0014) and liraglutide (L: -0.0355 ± 0.0011) groups were significantly different (p <0.001; Figure 4A). AUC insulin did not differ between the treatment groups (p = 0.73), although insulin levels were a tendency higher at the first time points after glucose administration and significantly lower at time points 40 and 50 minutes after glucose administration in liraglutide- vs. placebo-treated animals (Figure 4B).

### Effects on gastric emptying and organ weights

Liraglutide-treated animals showed a general tendency of lower absolute organ weights, while relative organ weights tended to be increased. Absolute pancreas weight of liraglutide- and placebo-treated pigs was 104.9 ± 3.7 g and 118.1 ± 8.4 g (p = 0.260). Relative pancreas weight was significantly increased in liraglutide-treated vs. placebo-treated animals (0.165 ± 0.005 and 0.127 ± 0.005, p < 0.001). At necropsy, the stomach of 12-hour-fasted liraglutide-treated animals still contained large amounts of food, while the stomach of placebo-treated animals was empty or filled with liquid.

### Reduced total alpha-cell volume, total beta-cell volume and beta-cell proliferation, but unaltered acinus-cell proliferation

**Figure 5:**
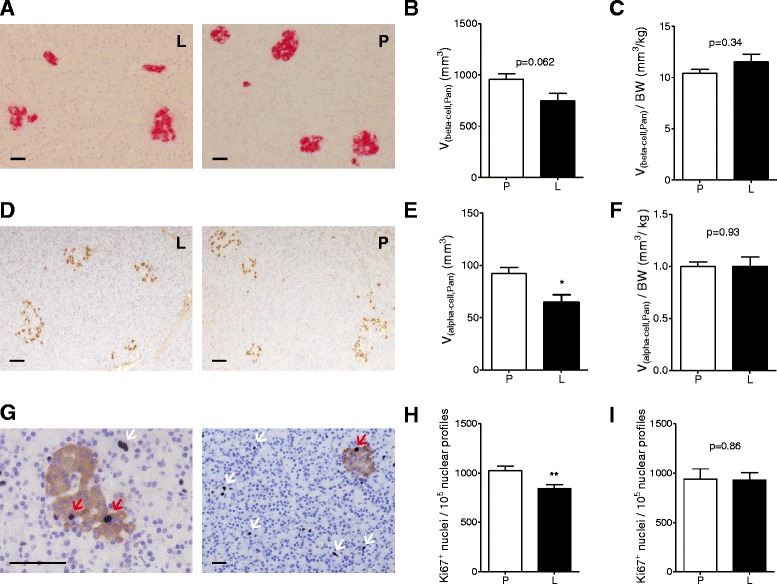
**Quantitative-stereological analyses of pancreata from liraglutide- and placebo-treated GIPR** ^**dn**^ **transgenic pigs.** Representative histological pancreas sections of **(A)** immunohistochemical staining for insulin and **(D)** glucagon as well as **(G)** double immunohistochemical staining for Ki67 (blue) and insulin (brown) of liraglutide- (L) and placebo-treated (P) GIPR^dn^ transgenic pigs, red arrow: proliferating beta-cell, white arrow: proliferating acinus-cell, scale bar = 50 μm. **(B)** Total beta-cell volume [V_(beta-cell, Pan)_] and **(C)** total beta-cell volume related to body weight [V_(beta-cell, Pan)_/BW] as well as **(E)** total alpha-cell volume [V_(alpha-cell, Pan)_] and **(F)** total alpha-cell volume related to body weight [V_(alpha-cell, Pan)_/BW]. **(H)** Beta-cell proliferation rate and **(I)** acinus-cell proliferation rate. Data are means ± SEM, *: p < 0.05, **: p < 0.01.

Quantitative-stereological analyses of pancreatic sections immunohistochemically stained for insulin (Figure 5A) revealed a reduced total beta-cell volume in liraglutide- vs. placebo-treated pigs with borderline significance (745.70 mm^3^ vs. 957.82 mm^3^, p = 0.062, Figure 5B). However, the total beta-cell volume-to-body weight ratio was not different between liraglutide- and placebo-treated animals (p = 0.34, Figure 5C). Total alpha-cell volume was reduced by 30% in liraglutide- compared to placebo-treated pigs (p < 0.05, Figure 5E). However, no differences were visible when alpha-cell mass was related to body weight (p = 0.93, Figure 5F). Proliferation rate of beta-cells was significantly reduced by 17% in liraglutide- vs. placebo-treated pigs (p < 0.01, Figure 5H). Acinus-cell proliferation did not differ between liraglutide- and placebo-treated animals (p = 0.86, Figure 5I). Evaluation of H&E stained pancreatic sections did not reveal histological abnormalities in the endocrine or exocrine pancreas of liraglutide-treated animals.

### Reduced activation of the growth related insulin signaling pathway in skeletal muscle

**Figure 6:**
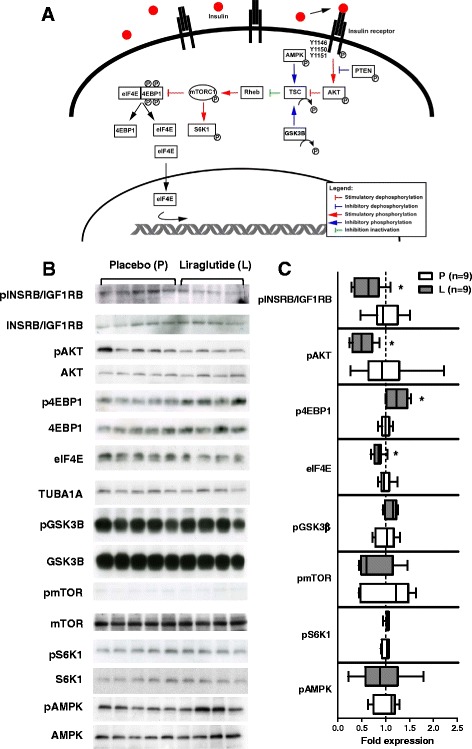
**Western blot analysis of the mTOR mediated insulin-stimulated pathway. (A)** Overview of the growth-regulating mTOR signaling pathway. **(B)** Western blot analyses of protein extracted from skeletal muscle showing blots of phosphorylated (upper panel) and not phosphorylated (lower panel) up- and down-stream regulators of the mTOR pathway in liraglutide- (left side of the blot) and placebo-treated (right side of the blot) GIPR^dn^ transgenic pigs. For elF4E not phosphorylated molecule (upper panel) is referred to tubulin (lower panel). **(C)** Quantitative analysis of Western blot signals, results are expressed as ratio of phosphorylated to not phosphorylated regulator and for eIF4E as absolute fold expression, related to the mean value of the placebo group, respectively, and presented as box plots with median, n = number of animals investigated, black plots = liraglutide-treated animals, white plots = placebo-treated animals, * = p < 0.05.

Western blot analyses investigating the growth regulating mammalian target of rapamycin (mTOR) complex including up- and downstream pathways revealed decreased phosphorylation of the INSRB/IGF1RB and AKT and increased phosphorylation of eukaryotic initiation factor 4E binding protein (4EBP1) in liraglutide- vs. placebo-treated pigs (p < 0.05, Figure 6). The phosphorylation status of mTOR, AMP-activated protein kinase (AMPK), S6 kinase 1 (S6K1) and glycogen synthase kinase 3 beta (GSK3β) was not affected by liraglutide treatment. The amount of the eukaryotic translation initiation factor 4E (elF4E) was significantly reduced in liraglutide- vs. placebo-treated animals (p < 0.05, Figure 6).

## Discussion

The present study evaluated the effect of a 90-day liraglutide treatment on growth, food intake, glycemic control, as well as endocrine and exocrine pancreas in adolescent transgenic pigs showing key characteristics of a pre-diabetic state. Liraglutide was the GLP1R agonist of choice as it improved glycemic control in adult type 2 diabetic patients more effectively than exenatide [4,5].

In contrast to clinical trials with adult type 2 diabetic patients that report weight loss of few percent [3], the body weight gain reducing effect in adolescent pigs was markedly more pronounced. This may be due to several factors: First, plasma liraglutide levels of pigs used in the present study were higher than in humans treated with the same dose (1.2 mg), most likely due to the higher overall bioavailability of liraglutide in pigs [2]. Additionally, the GIPR^dn^ transgenic pig model shows a compensatory enhanced effect to exendin-4 during stimulation tests and thereby seems to be especially sensitive to GLP1 [7]. Furthermore it has to be taken into account that adolescent organisms could be more sensitive to GLP1R agonist treatment compared to adult subjects. Human clinical studies investigating the influence of liraglutide in adolescents are rare and only outcomes from small clinical trials are available to date, despite of a steadily increasing prevalence of type 2 diabetes in youth [6,31]. One study in adolescent type 2 diabetic subjects treated with liraglutide did not detect alterations in body weight [32] while a case report noted a slight weight increase after 2 months of liraglutide treatment [33]. Due to the relatively slow growth of human adolescents, effects of liraglutide on growth and weight gain are unlikely to be detected during short treatment periods of two months or less. In contrast, growth and weight gain of young pigs is fast and continuous [34], thus providing a sensitive test system for detecting growth related drug effects within a reasonable period of time. Nevertheless translation of findings of the present study to adolescent type 2 diabetic patients is limited by the fact that the dose of liraglutide was higher than in previous studies in pigs [35,36], resulting in higher plasma liraglutide levels compared to human trials [2]. Further, the pigs used in the present study were not obese, while most adolescent type 2 diabetic patients show concomitant obesity, which may further decrease liraglutide levels and modulate its biological effects.

The reduced food intake observed in liraglutide-treated pigs is probably due to the known ability of liraglutide to delay gastric emptying [13,35,36]. Notably, after a 12-hour fasting period at the end of the 90-day treatment, liraglutide-treated pigs still had large amounts of food in their stomach, excluding adaptation of stomach emptying against liraglutide treatment or even tachyphylaxis. Our observations in the juvenile pig model contrast studies in young rats suggesting delayed gastric emptying as an acute effect of liraglutide treatment [37]. Reduced food intake may lead to a condition of undernutrition in the period of rapid growth as supported by reduced serum total protein and albumin concentrations. Significantly reduced serum phosphate concentrations in liraglutide-treated pigs may point to an effect on bone growth and/or metabolism, which is in line with the smaller appearance of the liraglutide-treated animals. In addition, liraglutide significantly reduced feeding efficiency, indicating a lower capability to dispose the food taken up. Studies in rodents suggested that liraglutide treatment increases energy expenditure [38,39], which remains to be determined in our pre-diabetic pig model.

Reduced insulin secretion in liraglutide-treated pigs corresponds to markedly decreased AUC glucose during MMGTT, most likely due to impaired gastric emptying and therefore delayed intestinal absorption of glucose [36,40]. Although liraglutide is a potent GLP1R agonist, it is likely that its insulinotropic effect depends on elevated glucose levels, as described for GLP1 [41]. Additionally, insulin sensitivity was – in accordance with findings in rodent and pig models [13,35,38,39,42] and with clinical studies [43] - significantly improved by liraglutide treatment, reducing the amount of insulin required for glucose disposal. This is also supported by the improved intravenous glucose tolerance observed in liraglutide-treated pigs. However, since our study did not include a weight-matched control group, we cannot exclude that the improved insulin sensitivity of the liraglutide-treated pigs is – at least in part – due to their reduced body weight gain.

The markedly reduced insulin secretion observed during MMGTT, which mimics normal food intake, may also have contributed to impaired body weight gain. This is supported by the fact that *INS*^C94Y^ transgenic pigs, which exhibit reduced insulin secretion, also show a markedly reduced body weight gain [44]. To address this hypothesis, we first performed holistic transcriptome analyses (available at Gene Expression Omnibus, record GSE56427) of *biceps brachii* muscle samples using Affymetrix custom Gene ST arrays (SNOWBALLs520824F) as previously described [28]. Skeletal muscle was chosen as a primary target tissue for insulin actions [45] and a major constituent of pig body weight. Our analysis revealed some sex-dependent transcriptome differences but no effects related to liraglutide treatment/reduced insulin secretion were found. To determine the phosphorylation status of proteins of the insulin signaling pathway which are known to regulate growth and to be modulated by insulin and nutrient uptake, we performed Western blot analyses [46,47]. Liraglutide treatment and concomitantly reduced insulin levels resulted in reduced phosphorylation of INSRB/IGF1RB and AKT. Although there was no significant change in the phosphorylation status of mTOR, we observed increased phosphorylation of the downstream regulator 4EBP1, which releases eIF4E, allowing initiation of translation [46,48]. Nevertheless, the level of free eIF4E was reduced in liraglutide-treated animals. Taken together, these findings point to reduced activation of the insulin signaling pathway in skeletal muscle of liraglutide- compared to placebo-treated pigs [46-48].

In the present study, liraglutide treatment did not stimulate beta-cell proliferation or increase total beta-cell mass in comparison to placebo treatment. This contrasts rodent studies reporting an increase in beta-cell mass after liraglutide treatment, going along with enhanced beta-cell proliferation and/or decreased beta-cell apoptosis [12-14]. These studies showed that two weeks of liraglutide administration (200 μg/kg twice daily) in diabetic *db/db* mice causes a beta-cell volume increase of about 35%, accompanied by an enhanced beta-cell proliferation rate [12,14]. Two weeks of liraglutide treatment (200 μg/kg twice daily) resulted in a 30% increased beta-cell volume going along with improved beta-cell proliferation in normoglycemic *m/m* mice [14], and six weeks of liraglutide treatment (30 μg or 150 μg twice daily) provoked increased beta-cell volume in Zucker diabetic fatty rats [13]. However, findings in rodent studies show a broad variability, as the same animal strains treated with lower dosages or exhibiting a different metabolic status show unaltered or even reduced beta-cell volume after liraglutide treatment [12,13,38,42]. This suggests strong influence of variable parameters like strain, age, metabolic status and glycemic control, duration of treatment and dosage that may altogether modulate the effect of liraglutide treatment on the rodent organism [12]. Additionally, it has to be taken into account that the rodent pancreas shows higher capacity for beta-cell proliferation compared to the human pancreas [20,21], and that – due to the shorter half-life of liraglutide in rodents (4-8 h) vs. humans (13-15 h) and pigs (14 h) [2] – liraglutide was administered twice daily in rodents. Studies investigating the effect of GLP1R agonists in non-rodent models like pigs or monkeys are rare, however provide more consistent results. In pancreaticoduodenectomized Yucutan miniature pigs that received an infusion of a marginal mass of pancreatic islets into the portal circulation, a six-week administration of liraglutide (20 μg/kg maintenance dose) could improve metabolic function of the animals, but the quantitative proportion of beta-cells in the transplanted islets did not differ between liraglutide- and placebo-treated animals [11]. Additionally, long-term studies in *Cynomolgus* monkeys (52-week duration using a dose up to 5 mg/kg/day) resulted in unaltered endocrine cell mass and proliferation rate [9,10].

Liraglutide-treated pigs of the present study exhibited only a moderate increase in plasma glucose after food intake, reducing the demand for insulin secretion and beta-cell proliferation. When related to body weight, total beta-cell mass was not different between liraglutide- and placebo-treated pigs which is in accordance with previous reports describing a linear correlation between beta-cell mass and body weight in rats and pigs [49,50].

Total alpha-cell mass was significantly reduced in liraglutide- vs. placebo-treated GIPR^dn^ transgenic pigs, while alpha-cell distribution was not different between liraglutide/placebo treatment. This is not in agreement with the increase of alpha-cell mass and abnormal alpha-cell distribution seen in pancreata of type 2 diabetic patients after incretin therapy [25]. The authors suggested reduced glucagon secretion induced by GLP1R agonist treatment as a fundamental reason [25]. However, in our study glucagon levels in liraglutide-treated pigs did not differ from those of placebo-treated pigs. Additionally, our finding reproduces the reduced alpha-cell mass observed in normoglycemic mice after liraglutide treatment [42].

Recent studies raised concern that incretin treatment might be accompanied by a proliferative effect on the exocrine pancreas, leading to histological changes, pancreatitis and an increased risk of pancreatic cancer in the long run [22-25,42], although these observations were not confirmed by other reports [9,17,51-54]. As the relative pancreas weight of liraglutide-treated pigs was increased compared to placebo-treated animals, we determined the proliferation rate of acinus-cells in the exocrine pancreas, but did not detect differences following liraglutide treatment. Therefore we exclude a proliferative effect of liraglutide on the exocrine pancreas in the GIPR^dn^ transgenic pig model. This is in line with studies investigating *Cynomolgus* monkeys and rats under incretin treatment that showed no differences in exocrine pancreas proliferation rate [9,17], and supported by the ADA/EASD/IDF consensually stating no need for modification of the current treatment recommendations on the basis of the current study data [55].

## Conclusions

The GIPR^dn^ transgenic pig model recapitulated principal clinical effects of liraglutide observed in type 2 diabetic patients. However, the reduction of body weight gain seen in adolescent pigs was more pronounced than the body weight-reducing effect after treatment of adult patients. Total alpha- and beta-cell mass was reduced in liraglutide- vs. placebo-treated animals, but not when related to body weight. Liraglutide treatment neither stimulated beta-cell proliferation in the endocrine pancreas nor acinus-cell proliferation in the exocrine pancreas after a 90-day treatment trial in our adolescent pre-diabetic pig model.

## Additional files

Additional file 1: Table S1. Composition of *ad libitum* offered standard pig diets produced by Zimmerer Werk, Landshut, Germany. Additional file 2: Table S2. Antibodies employed for Western blot analyses and their dilutions, all antibodies from Cell Signaling, Frankfurt, Germany. Additional file 3: Table S3. Clinical-chemical parameters in GIPR^dn^ transgenic pigs.

## Abbreviations

AKT: Protein kinase B
AMPK: AMP-activated protein kinase
AUC: Area under the curve
4EBP1: Eukaryotic initiation factor 4E binding protein
elF4E: Eukaryotic translation initiation factor 4E
GIP: Glucose-dependent insulinotropic polypeptide
GIPR: Glucose-dependent insulinotropic polypeptide receptor
GIPR^dn^: Dominant-negative GIPR
GLP1: Glucagon-like peptide-1
GLP1R: Glucagon-like peptide-1 receptor
GSK3B: Glycogen synthase kinase 3beta
HOMA-IR: Homeostasis model assessment-estimated insulin resistance
ISI_(Matsuda)_: Insulin sensitivity index according to Matsuda, IGF1R, Insulin-like growth factor 1 receptor
INSR: Insulin receptor
IVGTT: Intravenous glucose tolerance test
MMGTT: Mixed meal glucose tolerance test
mTOR: Mammalian target of rapamycin
S6K1: S6 kinase 1
