# Supplementary material for: Effects of the glucagon-like peptide-1 receptor agonist liraglutide in juvenile transgenic pigs modeling a pre-diabetic condition
Source: J Transl Med. 2015 Feb 25;13:73. doi: 10.1186/s12967-015-0431-2 (PMC4362632; doi:10.1186/s12967-015-0431-2)
Supplement: Additional file 1: Table S1. — Composition of ad libitum offered standard pig diets produced by Zimmerer Werk, Landshut, Germany. [file 12967_2015_431_MOESM1_ESM.docx]

**Supplementary Table 1: Composition of *ad libitum* offered standard pig diets produced by Zimmerer Werk, Landshut, Germany**

|  | **Ferkelstarter UNI**  **(piglets up to 25 kg)** | **Zuchtschwein Getreidemischung UNI**  **(growing and adult pigs)** |
| --- | --- | --- |
| **MJ ME/kg** | 13.1 | 10.9 |
| **Crude protein %** | 17.5 | 13.6 |
| **Crude fat %** | 2.9 | 3.2 |
| **Crude ash %** | 5.3 | 5.9 |
| **Crude fiber %** | 3.5 | 7.9 |
| **Calcium %** | 0.7 | 0.9 |
| **Phosphorus %** | 0.6 | 0.6 |
| **Sodium %** | 0.2 | 0.2 |
| **Magnesium %** | 0.2 | 0.3 |
