# Supplementary material for: Effects of the glucagon-like peptide-1 receptor agonist liraglutide in juvenile transgenic pigs modeling a pre-diabetic condition
Source: J Transl Med. 2015 Feb 25;13:73. doi: 10.1186/s12967-015-0431-2 (PMC4362632; doi:10.1186/s12967-015-0431-2)
Supplement: Additional file 2: Table S2. — Antibodies employed for Western blot analyses and their dilutions, all antibodies from Cell Signaling, Frankfurt, Germany. [file 12967_2015_431_MOESM2_ESM.docx]

**Supplementary Table 2: Antibodies employed for Western blot analyses and their dilutions, all antibodies from Cell Signaling, Frankfurt, Germany**

| **Antigen** | **Antibody** | **Host** | **Dilution** |
| --- | --- | --- | --- |
| p-GSK3β | #9322 | rabbit | 1:2000 |
| GSK3β | #9315 | rabbit | 1:2000 |
| p-Akt1 | #4060 | rabbit | 1:2000 |
| Akt1 | #4691 | rabbit | 1:2000 |
| p-mTOR | #5536 | rabbit | 1:1000 |
| mTOR | #2983 | rabbit | 1:1000 |
| p-AMPKα | #2535 | rabbit | 1:2000 |
| AMPKα | #2532 | rabbit | 1:2000 |
| p-4EBP1 | #2855 | rabbit | 1:2000 |
| 4EBP1 | #9644 | rabbit | 1:2000 |
| eIF4E | #9742 | rabbit | 1:2000 |
| p-INSRB/IGF1RB | #3024 | rabbit | 1:1000 |
| INSRB/IGF1RB | #3025 | rabbit | 1:1000 |
| p-p70S6K | #9205 | rabbit | 1:2000 |
| p70S6K | #2708 | rabbit | 1:2000 |
| TubA1A | #2521 | rabbit | 1:5000 |
