# Supplementary material for: Effects of the glucagon-like peptide-1 receptor agonist liraglutide in juvenile transgenic pigs modeling a pre-diabetic condition
Source: J Transl Med. 2015 Feb 25;13:73. doi: 10.1186/s12967-015-0431-2 (PMC4362632; doi:10.1186/s12967-015-0431-2)
Supplement: Additional file 3: Table S3. — Clinical-chemical parameters in GIPRdn transgenic pigs. [file 12967_2015_431_MOESM3_ESM.docx]

**Supplementary Table 3: Clinical-chemical parameters in GIPR^dn^ transgenic pigs**

Clinical-chemical parameters in liraglutide- (L, n=9) and placebo-treated (P, n=9) GIPR^dn^ transgenic pigs prior to (8 weeks of age) and during the 90-day liraglutide/placebo treatment period. Longitudinal data were square-root transformed to approximate normal distribution. Transformed data of clinical-chemical analyses were evaluated by ANOVA (General Linear Models; SAS 8.2) taking the fixed effects of Group, Age, and the interaction Group*Age into account.

| **Parameter** | **Group** | **Age** | | | | | | | | | | | | **Analysis of variance** | | |
| --- | --- | --- | --- | --- | --- | --- | --- | --- | --- | --- | --- | --- | --- | --- | --- | --- |
|  |  | **8 weeks** | | | **12 weeks** | | | **16 weeks** | | | **20 weeks** | | | **Group** | **Age** | **G × A** |
| **Leucocytes** | L | 14.0 | ± | 0.7 | 13.3 | ± | 0.8 | 18.1 | ± | 1.0 | 16.6 | ± | 0.8 | n.s. | *** | n.s. |
| [x 10^9^/l] | P | 15.0 | ± | 1.1 | 12.4 | ± | 1.4 | 16.6 | ± | 1.2 | 16.1 | ± | 1.1 |  |  |  |
|  |  |  |  |  |  |  |  |  |  |  |  |  |  |  |  |  |
| **Glucose** | L | 89.9 | ± | 2.8 | 95.7 | ± | 4.1 | 82.9 | ± | 1.4 | 81.1 | ± | 1.9 | n.s. | * | n.s. |
| [mg/dl] | P | 89.7 | ± | 2.1 | 86.5 | ± | 6.2 | 80.9 | ± | 3.3 | 88.3 | ± | 4.0 |  |  |  |
|  |  |  |  |  |  |  |  |  |  |  |  |  |  |  |  |  |
| **Bilirubin** | L | 2.2 | ± | 0.4 | 2.4 | ± | 0.5 | 1.9 | ± | 0.2 | 2.0 | ± | 0.3 | n.s. | n.s. | n.s. |
| [µmol/l] | P | 2.3 | ± | 0.3 | 4.1 | ± | 0.9 | 2.2 | ± | 0.4 | 1.8 | ± | 0.3 |  |  |  |
|  |  |  |  |  |  |  |  |  |  |  |  |  |  |  |  |  |
| **Urea** | L | 2.9 | ± | 0.3 | 3.7 | ± | 0.4 | 3.1 | ± | 0.5 | 3.4 | ± | 0.3 | n.s. | n.s. | n.s. |
| [mmol/l] | P | 2.9 | ± | 0.2 | 3.7 | ± | 0.3 | 2.9 | ± | 0.3 | 2.6 | ± | 0.3 |  |  |  |
|  |  |  |  |  |  |  |  |  |  |  |  |  |  |  |  |  |
| **Creatinine** | L | 44.0 | ± | 1.6 | 58.4 | ± | 5.9 | 101.3 | ± | 3.6 | 100.1 | ± | 2.3 | n.s. | *** | n.s. |
| [µmol/l] | P | 49.3 | ± | 2.4 | 61.2 | ± | 3.9 | 96.8 | ± | 4.3 | 105.6 | ± | 5.3 |  |  |  |
|  |  |  |  |  |  |  |  |  |  |  |  |  |  |  |  |  |
| **Total protein** | L | 46.2 | ± | 1.1 | 46.9 | ± | 0.8 | 53.6 | ± | 1.2 | 51.2 | ± | 1.0 | *** | *** | n.s. |
| [g/l] | P | 46.1 | ± | 0.7 | 51.5 | ± | 1.3 | 58.5 | ± | 1.4 | 55.9 | ± | 1.2 |  |  |  |
|  |  |  |  |  |  |  |  |  |  |  |  |  |  |  |  |  |
| **Albumin** | L | 25.8 | ± | 1.3 | 29.3 | ± | 1.1 | 37.4 | ± | 1.2 | 26.6 | ± | 1.4 | * | *** | n.s. |
| [g/l] | P | 24.3 | ± | 1.0 | 33.0 | ± | 1.6 | 41.9 | ± | 1.3 | 31.7 | ± | 1.5 |  |  |  |
|  |  |  |  |  |  |  |  |  |  |  |  |  |  |  |  |  |
| **Sodium** | L | 137.9 | ± | 0.6 | 140.1 | ± | 0.9 | 141.3 | ± | 0.8 | 140.8 | ± | 0.6 | n.s. | *** | n.s. |
| [mmol/l] | P | 138 | ± | 0.7 | 141.8 | ± | 0.7 | 143.2 | ± | 0.9 | 140.8 | ± | 0.5 |  |  |  |
|  |  |  |  |  |  |  |  |  |  |  |  |  |  |  |  |  |
| **Potassium** | L | 4.1 | ± | 0.1 | 4.8 | ± | 0.2 | 4.9 | ± | 0.2 | 4.7 | ± | 0.4 | n.s. | * | n.s. |
| [mmol/l] | P | 4.4 | ± | 0.2 | 4.7 | ± | 0.1 | 4.8 | ± | 0.1 | 4.1 | ± | 0.1 |  |  |  |
|  |  |  |  |  |  |  |  |  |  |  |  |  |  |  |  |  |
| **Chloride** | L | 98.2 | ± | 0.8 | 97.6 | ± | 0.8 | 99.0 | ± | 0.7 | 97.9 | ± | 1.0 | n.s. | n.s. | n.s. |
| [mmol/l] | P | 97.8 | ± | 0.6 | 98.0 | ± | 0.4 | 98.8 | ± | 0.4 | 98.2 | ± | 0.6 |  |  |  |
|  |  |  |  |  |  |  |  |  |  |  |  |  |  |  |  |  |
| **Calcium** | L | 2.3 | ± | 0.04 | 2.5 | ± | 0.04 | 2.5 | ± | 0.1 | 2.1 | ± | 0.1 | n.s. | *** | n.s. |
| [mmol/l] | P | 2.3 | ± | 0.05 | 2.5 | ± | 0.03 | 2.5 | ± | 0.03 | 2.3 | ± | 0.03 |  |  |  |
|  |  |  |  |  |  |  |  |  |  |  |  |  |  |  |  |  |
| **Phosphate** | L | 3.2 | ± | 0.1 | 3.0 | ± | 0.1 | 2.4 | ± | 0.1 | 2.6 | ± | 0.01 | ** | *** | * |
| [mmol/l] | P | 3.1 | ± | 0.1 | 3.4 | ± | 0.1 | 3.0 | ± | 0.1 | 2.7 | ± | 0.1 |  |  |  |
|  |  |  |  |  |  |  |  |  |  |  |  |  |  |  |  |  |
| **Iron** | L | 17.2 | ± | 1.8 | 26.2 | ± | 1.8 | 25.2 | ± | 2.5 | 20.0 | ± | 1.2 | n.s. | ** | n.s. |
| [µmol/l] | P | 18.9 | ± | 1.5 | 27.2 | ± | 3.5 | 24.3 | ± | 0.9 | 23.0 | ± | 1.6 |  |  |  |
|  |  |  |  |  |  |  |  |  |  |  |  |  |  |  |  |  |
| **Magnesium** | L | 1.1 | ± | 0.3 | 0.8 | ± | 0.01 | 0.9 | ± | 0.01 | 0.7 | ± | 0.1 | n.s. | n.s. | n.s. |
| [mmol/l] | P | 0.8 | ± | 0.03 | 0.9 | ± | 0.02 | 0.8 | ± | 0.03 | 0.7 | ± | 0.02 |  |  |  |
|  |  |  |  |  |  |  |  |  |  |  |  |  |  |  |  |  |
| **AST** | L | 30.6 | ± | 4.1 | 32.5 | ± | 5.3 | 22.1 | ± | 2.1 | 27.0 | ± | 2.3 | n.s. | n.s. | n.s. |
| [U/l] | P | 29.5 | ± | 3.2 | 33.6 | ± | 2.2 | 29.5 | ± | 1.7 | 33.9 | ± | 5.1 |  |  |  |
|  |  |  |  |  |  |  |  |  |  |  |  |  |  |  |  |  |
| **γGT** | L | 34.5 | ± | 3.7 | 40.9 | ± | 4.2 | 35.6 | ± | 3.2 | 37.2 | ± | 3.8 | n.s. | n.s. | n.s. |
| [U/l] | P | 32.8 | ± | 2.4 | 37.4 | ± | 4.8 | 36.6 | ± | 4.1 | 35.2 | ± | 4.8 |  |  |  |
|  |  |  |  |  |  |  |  |  |  |  |  |  |  |  |  |  |
| **AP** | L | 190.1 | ± | 9.2 | 185.6 | ± | 8.9 | 130.3 | ± | 7.3 | 109.1 | ± | 5.8 | ** | *** | n.s. |
| [U/l] | P | 157.9 | ± | 12.4 | 167.5 | ± | 7.7 | 119.1 | ± | 4.7 | 98.4 | ± | 3.5 |  |  |  |
